# Defining the Serratus Triangle: A Major Pillar for Male High-Definition Liposculpture

**DOI:** 10.1007/s00266-026-05794-3

**Published:** 2026-03-19

**Authors:** Mariachiara Fabbri, Ahmad Saad

**Affiliations:** 1 Residency Program in Plastic Surgery, Università Cattolica del “Sacro Cuore”, Largo A. Gemelli 8, 00168 Rome, Italy; 2 Plastic Surgeon in Private Practice at IMAGN Institute, Rambla de Catalunya, 53, 08007 Barcelona, Spain; 3 University of California, San Diego, CA USA

**Keywords:** Abdominal liposculpture, HD liposculpture, Male liposculpture, High definition, Aesthetic surgery, Serratus triangle

## Abstract

**Background:**

High-definition (HD) liposculpture aims to enhance male body aesthetics by sculpting natural muscular definition. However, one of the most commonly overlooked areas in the torso is the serratus triangle, whose definition is essential to achieve a harmonious transition between the pectoralis major, latissimus dorsi, and lateral abdominal wall. Failure to address this area can result in an unnatural or incomplete appearance.

**Objectives:**

To describe a refined surgical approach for defining the serratus triangle during male HD liposculpture and to evaluate its efficacy, safety, and reproducibility in a consecutive series of patients.

**Methods:**

A retrospective study of 200 consecutive male patients who underwent HD liposculpture including serratus triangle definition between 2020 and 2024. The technique involves deep and superficial liposuction along the serratus lines, with selective use of the Renuvion® technology in cases with nipple-areola complex ptosis.

**Results:**

All 200 patients completed a minimum follow-up of 8 months. No major complications or cases of skin necrosis were observed. Ninety patients developed transient fibrosis in the treated area, which resolved within 6–8 weeks with compliance to the postoperative exercise and massage protocol.

**Conclusions:**

Defining the serratus triangle is a simple yet often-overlooked step that significantly enhances the natural, athletic look of male HD liposculpture. This technical refinement is safe, reproducible, and can be seamlessly integrated into existing HD protocols to improve aesthetic outcomes.

**Level of Evidence III:**

This journal requires that authors assign a level of evidence to each article. For a full description of these Evidence-Based Medicine ratings, please refer to the Table of Contents or the online Instructions to Authors www.springer.com/00266.

**Supplementary Information:**

The online version contains supplementary material available at 10.1007/s00266-026-05794-3.

## Introduction

Since the introduction of high-definition (HD) liposculpture [1–6], the demand for sculpted, athletic abdominal aesthetics has steadily increased worldwide. According to the latest ISAPS Global Statistics [7], liposuction remains one of the most popular cosmetic surgical procedures, with over 2 million procedures performed globally every year. Among male patients, liposuction ranks as the fourth most requested surgical procedure, reflecting a growing interest in achieving a more defined and athletic torso. A review of the existing literature on male abdominal etching shows a consistent focus on enhancing the rectus abdominis inscriptions, linea alba, and semilunar lines [8–11]. However, there is a striking lack of discussion regarding a small yet crucial area for achieving a balanced, natural masculine torso: the serratus triangle. This area is frequently overlooked during male body contouring procedures, and its omission can result in outcomes that appear incomplete, artificial, or disharmonious.

**Fig. 1:**
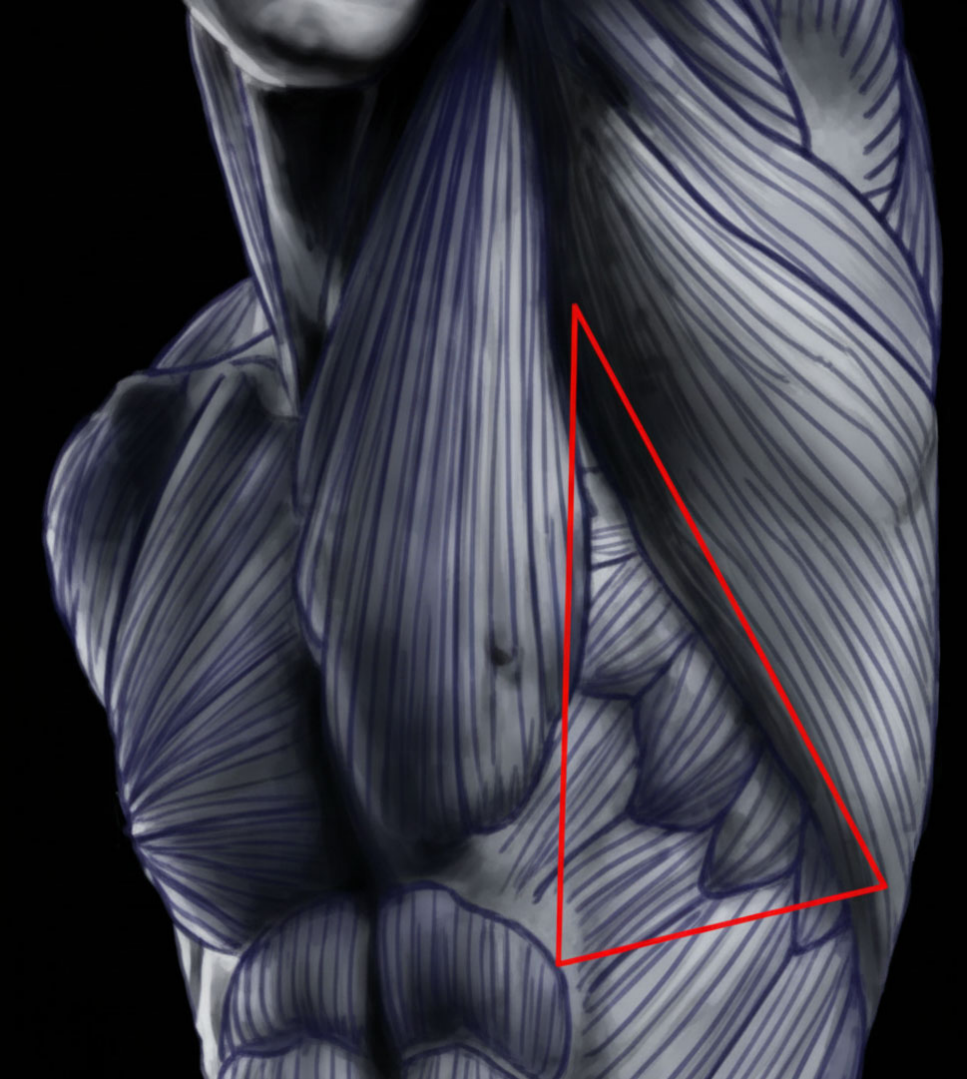
Identification of the serratus triangle (Image created with the help of AI)

Anatomically, the serratus triangle (Fig. 1) is bordered by:

- Anteriorly by the oblique edge of the pectoralis major
- Posteriorly by the latissimus dorsi
- Superiorly by the dome of the axilla
- Inferiorly in continuation with the lateral abdominal wall (the external oblique muscle)
- On top of the serratus anterior muscles

The aim of this article is to highlight the aesthetic importance of this often-overlooked area through presenting our personal technique and sharing our clinical experience from a consecutive series of male patients undergoing HD liposculpture.

## Materials and Methods

A retrospective study was conducted of 200 consecutive male patients treated between 2020 and 2024 by the same senior surgeon.

The study followed the Declaration of Helsinki; all patients signed informed consent.

### Patient Selection

All cases included in the review represent male patients seeking HD liposculpture, in whom the serratus triangle was always addressed as an integral step to ensure a balanced and natural result. For this reason, inclusion and exclusion criteria mirrored standard abdominal etching:

#### Inclusion Criteria

- Age >18 years
- BMI <30 kg/m^2^
- ASA ≤2

#### Exclusion Criteria

- Poorly controlled comorbidities (diabetes and hypertension)
- Active smokers (required cessation ≥5 week preoperative and ≥6-week postoperative)
- Significant skin laxity that necessitates skin excision (only mild–moderate acceptable)
- Nipple areola complex (NAC) below the inframammary fold IMF [12]
- Poor skin quality

### Materials and Technologies

- Tumescent fluid composed of superwet technique with a solution consisting of 1000 mL of 0.9% saline, 20 mL of 1% lidocaine, and 1 mL of epinephrine at a concentration of 1:1000.
- Power-assisted liposuction (PAL) by MicroAire® (Inc, Charlottesville, VA)
- 5-mm basket cannula or PAL HD straight cannula by MicroAire® (Inc, Charlottesville, VA)
- Renuvion® (Apyx Medical, Florida)

### Operative Technique

Patient marking is performed in the preoperative area in a standing position. The inferior and oblique edges of the pectoralis major muscle are marked with the patient arms pressing down on the surgeon’s shoulders. The lateral edge of the latissimus dorsi (LD) is marked by asking the patient to put his hands on his waist and push in order to contract the muscle (Video 1).

Patient is positioned supine with arms abducted at approximately 45 degrees on adjustable arm boards. Access to the serratus triangle is gained through a small incision placed discreetly in the axillary fold, just posterior to the anterior axillary line, and also through a second incision within the inferior half of areola. Infiltration as above described, both into deep and superficial planes, is then carried out. To maximize the vasoconstrictive and analgesic effects of the solution, a waiting period of approximately 15 min is always observed before liposuction began (Video 2).

Power-assisted liposuction (PAL) (Microaire®, Inc, Charlottesville, VA) is then performed in two sequential phases. First, deep fat removal is carried out beneath the Scarpa fascia to debulk the area and allow proper contour transition. This is followed by a superficial sculpting phase where most superficial fat is removed within the marked serratus triangle and below the horizontal edge of the pectoralis major (Video 3). After aspiration, a phase of fat equalization is performed using the reciprocating PAL technology without suction, which helps smooth the superficial fat layer and eliminate any contour irregularities.

When indicated, in cases presenting with mild-to-moderate nipple-areolar complex NAC ptosis (as long as the nipple is above the IMF level), Renuvion® (Apyx Medical, Florida) technology is applied to promote lifting of the NAC on the pectoralis major. The device is set at 80% power with a helium gas flow of 2 L/min. Application of the technology is performed in a controlled, fan-shaped motion, maintaining a spacing of 2–3 cm between passes and an activation speed of 1–3 cm/sec to balance efficacy and minimize the risk of thermal injury. Typically, four passes are applied, while deliberately avoiding areas where superficial liposuction has been carried out to prevent additional risk of skin suffering (Video 4).

**Fig. 2:**
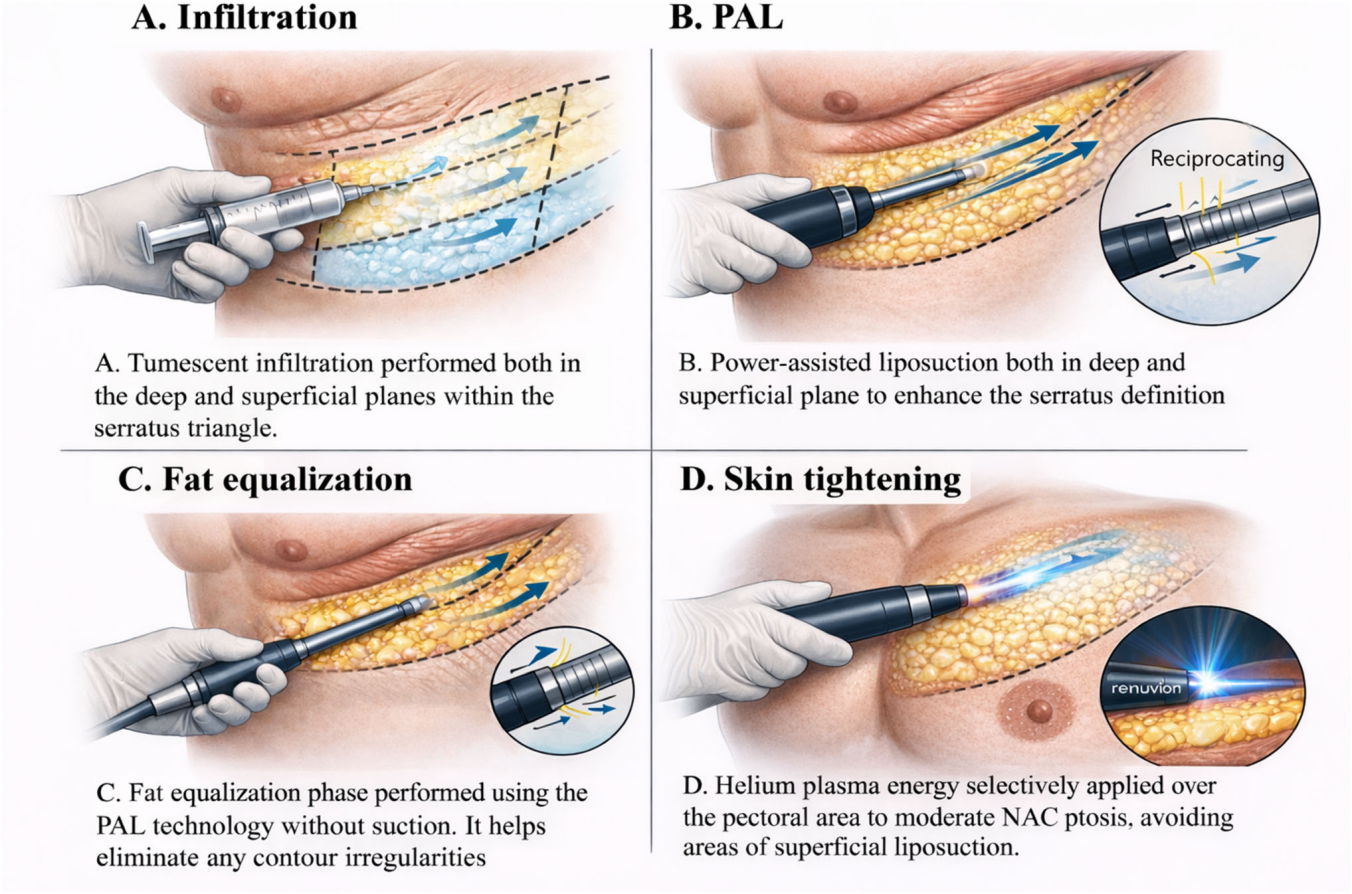
Four-step summary of serratus triangle sculpting (Image created with the help of AI)

For further clarity, we have created Figure 2, which provides a concise overview of all four main procedural steps.

Closure is performed with absorbable subcutaneous sutures and Steri-Strips, the area is dressed with compressive Epifoam and compressive garments to maintain contour and minimize dead space. No drains are used. Postoperatively, patients are monitored overnight to ensure proper fluid management and recovery, and they are discharged with oral antibiotics and analgesics, although NSAIDs are systematically avoided due to potential interference with helium plasma efficacy. All patients are instructed to wear the compressive garment day and night for three weeks to support edema reduction and skin redraping. Manual lymphatic drainage is started on the second postoperative day. To minimize the risk of postoperative fibrosis, patients are instructed from the first day to elevate their arms fully every 2 h. If restricted, they are asked to reach their maximum range of motion, extend by an additional centimeter, and hold the position for 20 s.

## Results

The mean patient age was of 41.5 years old (range 27–48 y.o.), and the mean BMI was of 26.9 (range 24, 9–29,8).

The average follow-up was of 19 months, with the minimum being 8 months and the maximum 4 years.

**Fig. 3:**
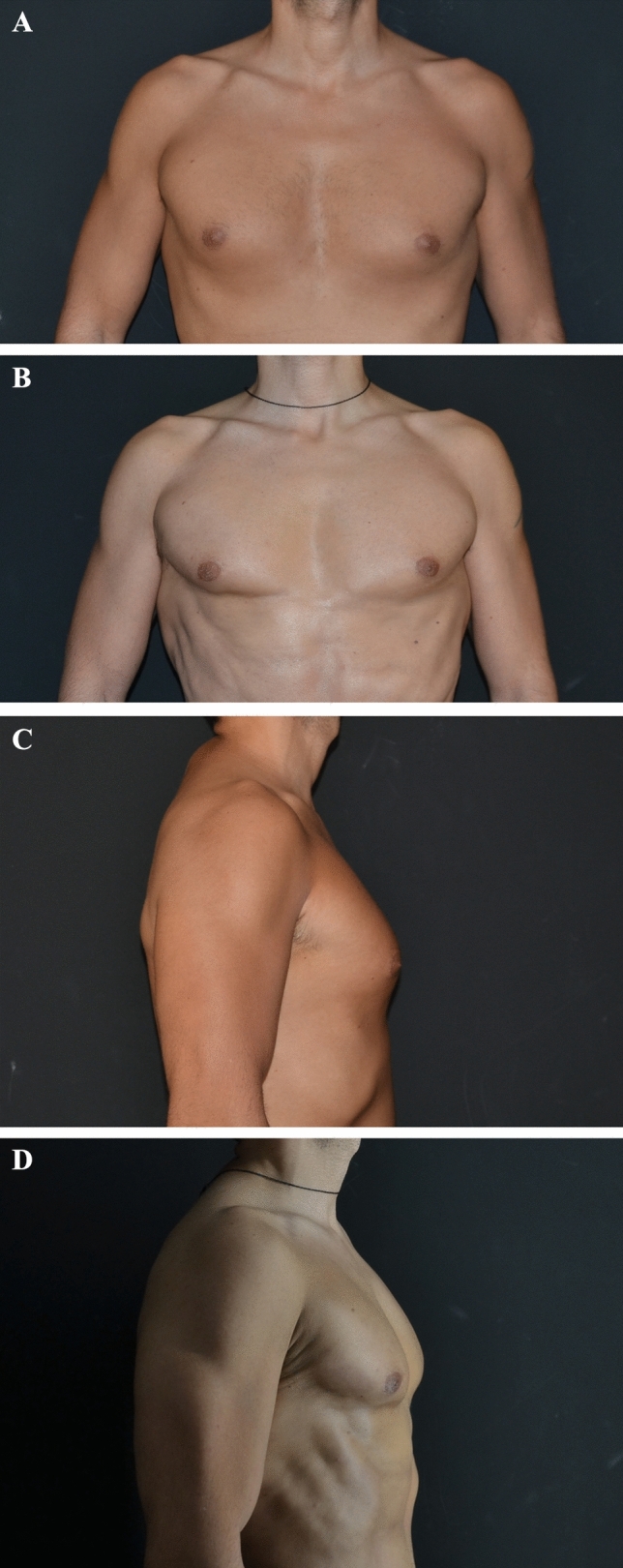
A 41-year-old patient **A** Preoperative frontal view **B** 1-year postoperative frontal view **C** Preoperative lateral view; and **D** 1-year postoperative lateral view

**Fig. 4:**
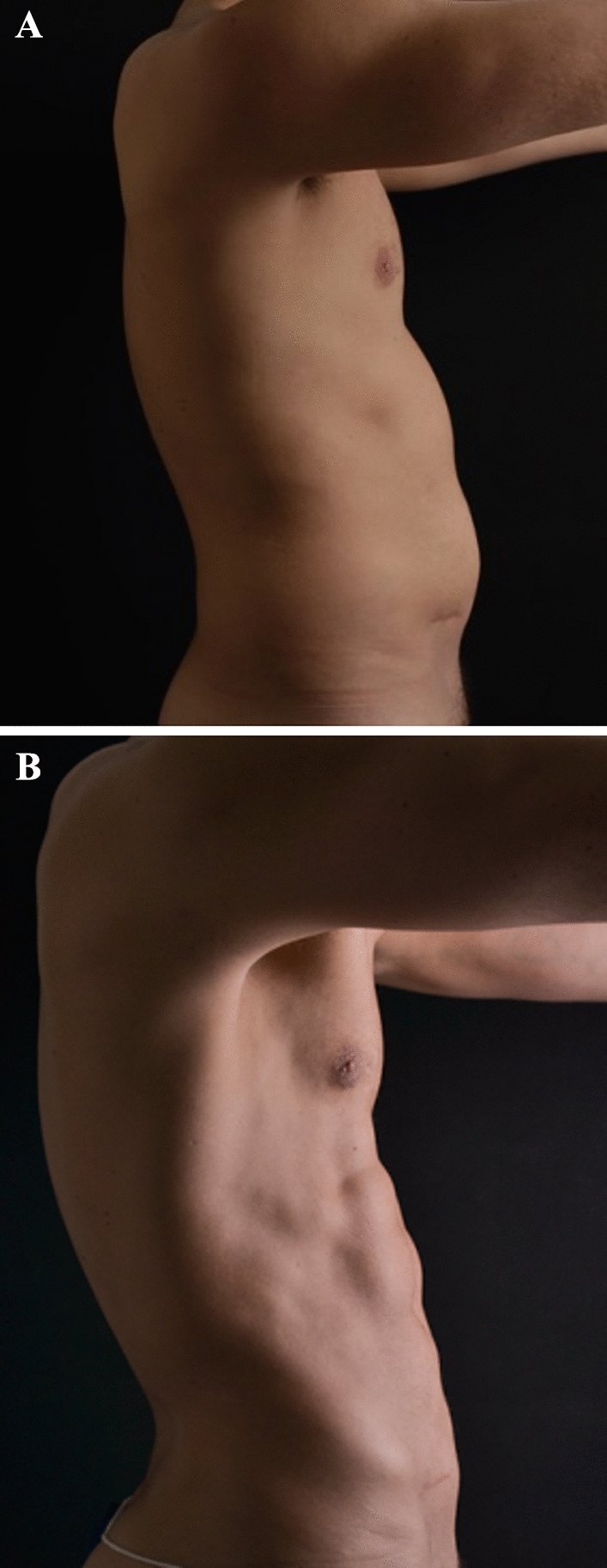
A 29-year-old patient **A** Preoperative lateral view and **B** 1.5-year operative lateral view

**Fig. 5:**
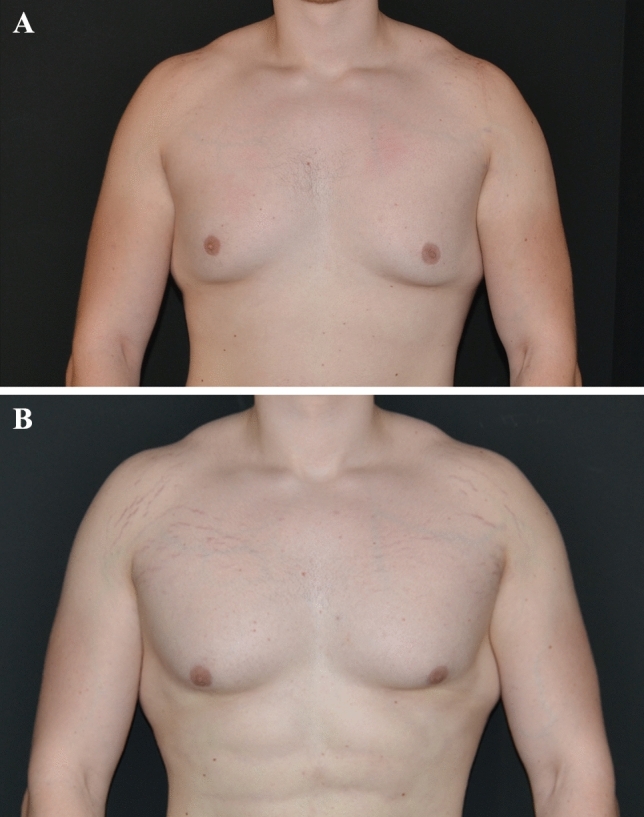
A 38-year-old patient **A** Preoperative frontal view and **B** 8-month operative frontal view

No cases of major life threatening and skin necrosis were observed. Transient fibrosis within the serratus triangle region developed in 90 patients (45%), typically resolving within 6 to 8 weeks with consistent adherence to massage protocols and the prescribed range-of-motion exercises. No cases of persistent fibrosis were observed beyond 3-month postoperative. Twenty-four patients (12%) presented with hemosiderosis along the demarcation line, treated in a conservative way and resolved over the following months and might take over a year for complete resolution. No revisions were required. No seromas were observed in this are, however, since all of these patients underwent a complete HD liposculture within abdomen and flanks, 24 of them (12%) did develop fluid collections in the lower abdomen which was treated through serial aspiration. Complications are described in Table 1. Figures 3, 4, and 5 are showing pre and postoperative pictures.

**Table 1:** Postoperative complications

Type of complications	Number and percentage
Transient fibrosis	90 patients (45%)
Permanent fibrosis	0 patients (0%)
Skin necrosis	0 patients (0%)
Hemosiderin	24 patients (12%)
Seroma collection at serratus triangle site	0 patients (0%)
Seroma collection at lower abdomen	24 patients (12%)

## Discussion

The popularity of high-definition liposculpture has grown exponentially in recent years, especially among male patients who seek an athletic, natural-looking body contour. While traditional abdominal etching techniques focus primarily on enhancing the rectus abdominis, linea alba, and semilunar lines, our experience suggests that neglecting adjacent anatomical transition zones can lead to results that appear incomplete or artificially segmented. In this context, the serratus triangle plays a pivotal role in achieving a harmonious and authentic male torso definition.

Despite its clear aesthetic relevance, the serratus triangle is rarely emphasized in the literature. Yet, this anatomical region defines the superolateral continuity between the chest and the upper abdominal wall. When properly sculpted, it frames the lateral thoracic silhouette and enhances the perception of a fit, athletic physique.

Our retrospective analysis of 200 consecutive cases demonstrates that the inclusion of this area in routine male HD liposculpture is safe, reproducible, and adds significant value to the final aesthetic outcome without increasing the risk of major complications. In our experience, the technical refinements described, including precise deep and superficial liposuction limited to well-defined lines, the use of PAL for better fat equalization, and the selective application of subdermal helium plasma for skin tightening, provide predictable and durable results.

A key element in preventing contour irregularities and ensuring smooth recovery is strict patient compliance with postoperative care instructions, especially the regimen of frequent arm elevation and early lymphatic massage. Fibrosis, although common in the early weeks, was transient in all patients and resolved with strict adherence to postoperative recommendations. This underlines the importance of clear patient education and active follow-up during the first postoperative weeks. Regarding the 12% incidence of hemosiderosis, these cases occurred mostly during the initial period of the study. Our observation indicates that proper infiltration of the superficial fat layer with tumescent fluid can significantly reduce its incidence.

Recently, alternative strategies for enhancing the oblique-serratus complex have been reported, particularly using ultrasound-guided fat grafting techniques. Flores González et al. (2023) [13] described ultrasound-guided intramuscular fat grafting of the serratus-external oblique muscle complex, demonstrating an immediate increase in muscle thickness after augmentation. Compared with our non-volumizing, sculpting-only approach, fat grafting provides the potential advantage of increasing local contour volume and improving three-dimensional muscular projection. However, this strategy typically involves increased operative time, a more complex procedural workflow (including real-time ultrasound guidance and targeted intramuscular injections), uneven fat take and some procedure-specific risks associated with intramuscular fat injection. Although small series such as Flores González et al. did not observe complications, larger studies of intramuscular fat grafting techniques have reported risks including fat embolism, infection, seroma, hematoma, fat necrosis, asymmetries, or the need for revisions [14]. In contrast, our approach focuses on selective deep and superficial liposuction along the serratus lines to enhance natural muscular definition without increasing tissue volume. This allows for a reproducible, less complex intervention with predictable outcomes and minimal additional risk. While volumization may be appropriate in select patients with low baseline muscular prominence, definition-only sculpting provides a reliable method for achieving a harmonious, athletic appearance in the majority of male patients.

A limitation of our analysis is its retrospective design and the lack of a control group. Future studies may benefit from including a blinded evaluations to further validate the impact of serratus triangle sculpting on overall aesthetic satisfaction. Nevertheless, our results strongly suggest that this technical nuance, while seemingly minor, can transform the final result from simply defined to genuinely natural and harmonious, aligning with current aesthetic ideals for the male abdomen.

## Conclusion

Defining the serratus triangle in male HD liposculpture is a safe, reproducible technical refinement that significantly improves the harmony and natural look of the torso, with minimal additional risk. This approach should be considered a standard part of male torso etching.

## Supplementary Information

Below is the link to the electronic supplementary material.

Supplementary file1:Preoperative marking demostration (MP4 8150 kb)

Supplementary file2:Infiltration phase (MP4 1835 kb)

Supplementary file3:Deep and superficial liposuction (MP4 4075 kb)

Supplementary file4:Skin tightening using RF device (MP4 1154 kb)
